# Self-compliance-improved resistive switching using Ir/TaO_*x*_/W cross-point memory

**DOI:** 10.1186/1556-276X-8-527

**Published:** 2013-12-17

**Authors:** Amit Prakash, Debanjan Jana, Subhranu Samanta, Siddheswar Maikap

**Affiliations:** 1 Thin Film Nano Tech. Lab., Department of Electronic Engineering, Chang Gung University, 259 Wen-Hwa 1st Rd., Kwei-Shan, Tao-Yuan 333, Taiwan

**Keywords:** RRAM, Cross-point, TaO_*x*_, Self-compliance

## Abstract

Resistive switching properties of a self-compliance resistive random access memory device in cross-point architecture with a simple stack structure of Ir/TaO_*x*_/W have been investigated. A transmission electron microscope and atomic force microscope were used to observe the film properties and morphology of the stack. The device has shown excellent switching cycle uniformity with a small operation of ±2.5 V and a resistance ratio of >100. The device requires neither any frorming-process nor current compliance limit for repeatable operation in contrast to conventional resistive random access memory devices. The effect of bottom electrode morphology and surface roughness is also studied. The improvement is due to the enhanced electric field at the nanotips in the bottom electrode and the defective TaO_*x*_ switching layer which enable controlled filament formation/rupture. The device area dependence of the low resistance state indicates multifilament formation. The device has shown a robust alternating current endurance of >10^5^ cycles and a data retention of >10^4^ s.

## Background

Resistive random access memory (RRAM) is the most promising candidate for the next-generation nonvolatile memory technology due to its simple structure, excellent scalability potential (<10 nm), long endurance, high speed of operation, and complementary metal-oxide-semiconductor (CMOS) process compatibility [1-7]. RRAM in cross-point architecture, in which top and bottom electrodes are placed at right angle to each other, is very attractive as it offers high-density integration with 4 *F*^2^, *F* being the minimum feature size area; three-dimensional (3D) stacking; and cost-effective fabrication [8,9]. Switching uniformity is one of the important properties which require practical realization of cross-point devices with large array size. So it is necessary to investigate the factors affecting switching uniformity. Various binary transition metal oxides such as HfO_*x*_[5,6,10-12], TiO_*x*_[13,14], TaO_*x*_[2,7,15-18], AlO_*x*_[19-21], ZrO_*x*_[22-24], WO_*x*_[25], etc. as a switching material are reported for RRAM application. Among them, recently, TaO_*x*_ has attracted much attention [26] owing to its superior material and switching properties such as having two stable phases [15], high thermal stability [18], small difference between the free energies of low and high resistance states [26], CMOS compatibility, long endurance [2], and high switching speed [7]. So far,a cross-point resistive switching memory device in an Ir/TaO_*x*_/W structure has not yet been reported.

In this study, self-compliance-limited and low-voltage-operated resistive switching behaviors with improved switching cycle uniformity in a simple resistive memory stack of Ir/TaO_*x*_/W in cross-point architecture are reported. The physical properties of switching stack and bottom electrode morphology have been observed by transmission electron microscope (TEM) and atomic force microscope (AFM) analyses. The improvement is due to the defective switching layer formation as well as the electric field enhancement at the nanotips observed in the bottom electrode surface which results in controlled and uniform filament formation/rupture. The self-compliance property shows the built-in capability of the device to minimize the current overshoot during switching in one resistance (1R) configuration. The device has shown an alternating current (ac) endurance of >10^5^ cycles and a data retention of >10^4^ s.

## Methods

**Figure 1:**
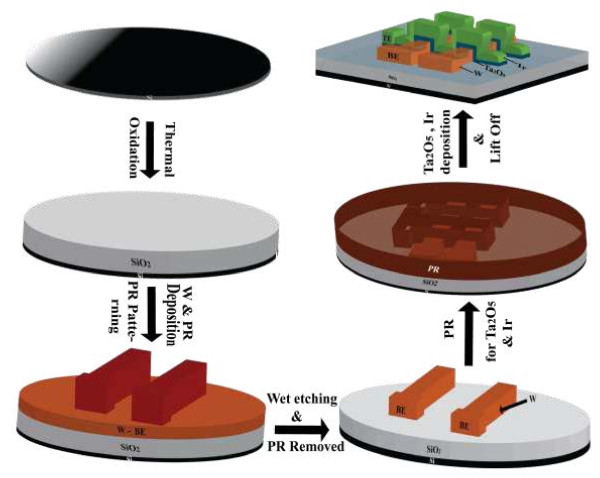
**Process flow of RRAM fabrication.** Process flow of the fabrication of TaO_*x*_*-*based cross-point resistive switching memory.

**Figure 2:**
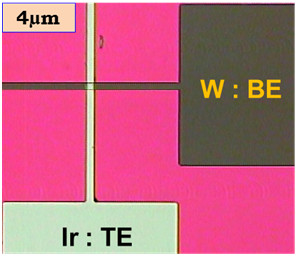
**Optical image of cross-point memory.** Optical microscope (OM) image of a single cross-point memory device.

A cross-point resistive memory stack in an Ir/TaO_*x*_/W structure have been fabricated on SiO_2_ (200 nm)/Si substrate. The fabrication steps are schematically depicted in Figure 1. A sputter-deposited W layer of approximately 250 nm was patterned using photolithography and wet etching methods in order to get W bottom electrode (BE) bars. A deposition power and pressure of 100 W and 5 mTorr, respectively, were used for the W layer deposition, and sizes (width) of W bars were between 4 and 50 μm. After an additional lithography patterning step for lift-off using a second mask at right angle to define top electrode (TE) bars, a TaO_*x*_ switching layer was deposited by an electron beam evaporator system using pure Ta_2_O_5_ granulates under a high vacuum of 2 × 10^−6^ Torr. To avoid any atmospheric oxidation/contamination effects on the TaO_*x*_ switching layer, an Ir layer of about 50 nm as TE was immediately deposited on the TaO_*x*_ layer using an Ir target by a sputtering system. The rf power and working pressure were 50 W and 5 mTorr, respectively, and the sizes of the TE bars were the same as those of the BE bars (4 to 50 μm). Finally, the lift-off process was performed to get the cross-point devices. The sizes of the cross-points were in the range of 4 × 4 to 50 × 50 μm^2^. An optical microscope image of such a cross-point with an area of 4 × 4 μm^2^ is shown in Figure 2. The TE and BE bars at right angles along with the contact pads are shown. The electrical characterizations have been performed using an Agilent 4156 C precision semiconductor parameter analyzer (Santa Clara, CA, USA) in voltage sweep mode at room temperature and ambient conditions. The voltage applied on TE and BE was electrically grounded during measurement.

## Results and discussion

**Figure 3:**
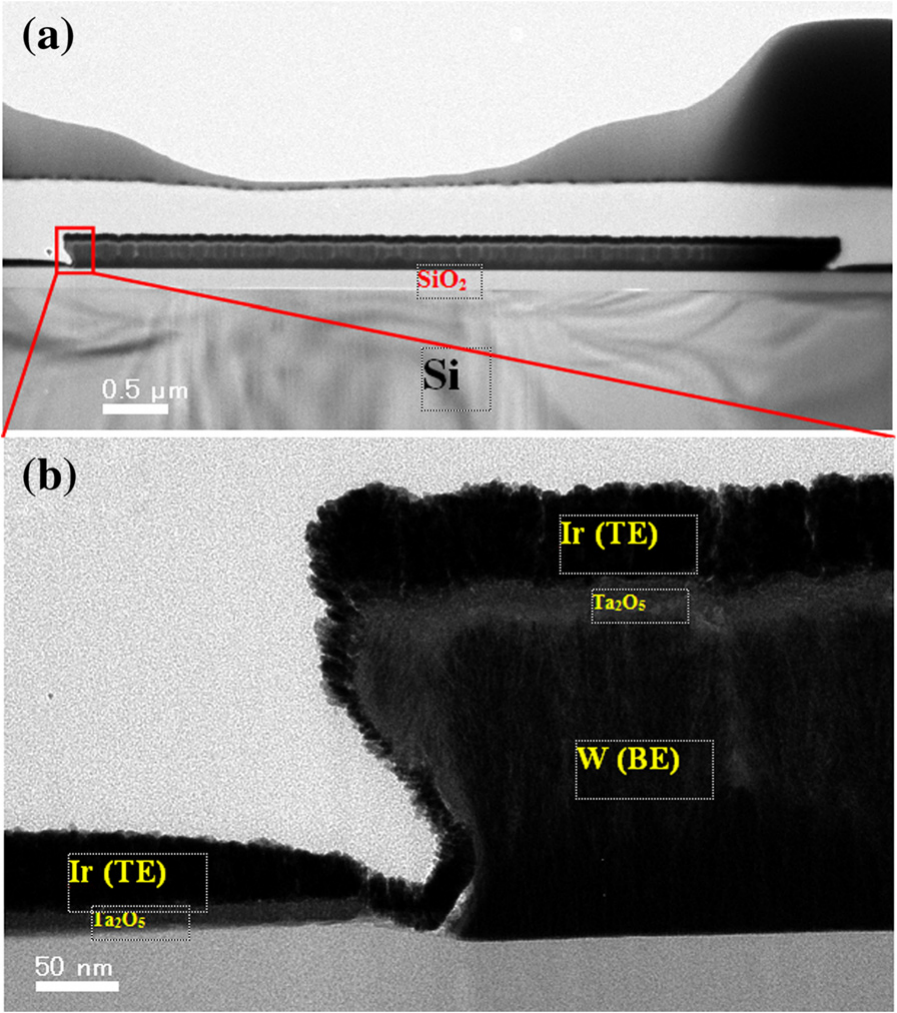
**TEM image of cross-point memory. (a)** TEM image and **(b)** sidewall view of cross-point resistive switching memory.

In order to confirm the fabricated RRAM device stack and film thickness, cross-sectional TEM images were acquired, as shown in Figure 3. The size of the cross-point is approximately 6 × 6 μm^2^ (Figure 3a). The TaO_*x*_ switching layer sandwiched between W (BE) and Ir (TE) metal electrodes is clearly visible, as shown in Figure 3b. The amorphous TaO_*x*_/WO_x_ layer thickness on the top of W BE is approximately 20 nm. The WO_*x*_ layer is formed during the fabrication process. The columnar growth of both metal electrodes is also evident in the TEM image. Further, the thickness of the stack layers is higher on the top of W BE than on the sidewall due to the sputtering deposition. The thickness of the TaO_*x*_/WO_*x*_ layer on the sidewall is approximately 10 nm, which is thinner than that of the top side (approximately 20 nm). This suggests that the conducting filament will be formed on the sidewall rather than the top side.

**Figure 4:**
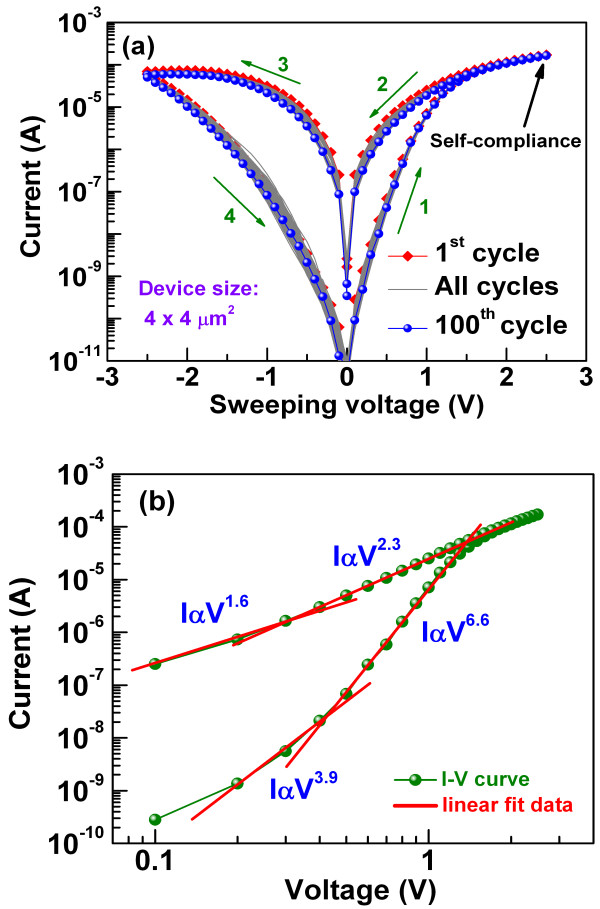
**Current–voltage switching and fitting curves. (a)** Consecutive excellent 100 *I*-*V* repeatable switching cycles and **(b)***I*-*V* fitting with TC-SCLC of self-compliance cross-point resistive switching memory devices.

The current–voltage (*I*-*V*) characteristics of the cross-point device in the Ir/TaO_*x*_/W structure are shown in Figure 4a. The initial resistance of the pristine device was higher than that of the high resistance state (HRS), and the first set voltage was almost similar to the subsequent set voltage (curve not shown here). Such type of forming step-free resistance memory devices is particularly attractive for practical realization because of its cost-effectiveness and reduction in circuit complexity. The BE morphology and smaller thickness of TaO_*x*_ on the sidewalls resulted this forming step-free behavior. The bipolar *I*-*V* curves of all the subsequent 100 consecutive direct current (dc) sweep cycles with highlighted 1st and 100th cycles are shown in Figure 4a. As no obvious difference between the first and the last cycle is observed, the device shows excellent switching cycle uniformity with tight distribution in low resistance state (LRS) and HRS. The small dispersion is required for large cross-point arrays. Further, unlike conventional RRAMs, this device does not require any current compliance limit for operation which indicates its built-in current overshoot reduction capability which is helpful in obtaining long pulse endurance without the use of a transistor as current limiter. The self-compliance behavior is due to the high bulk resistance of the device which resulted owing to the WO_*x*_ and electrically formed interface layer near the TE during the first cycle of device break-in [27]. Also, the *I*-*V* curve of the LRS is nonlinear and the resistance of the LRS is high (>100 kΩ). In order to investigate the current conduction mechanism in both LRS and HRS, the switching *I*-*V* curve in the positive-bias region is replotted in a log-log scale, as shown in Figure 4b. Two linear regions in LRS as well as in HRS were identified with the different slopes of 1.6 and 2.3, and 3.9 and 6.6, respectively. The slope values suggest that the conduction mechanism in both LRS and HRS is trap-controlled space-charge-limited conduction (TC-SCLC). At smaller voltage, traps are unfilled and hence current is small, whereas at higher voltage, the current increases fast (*I*∝*V*^2.3^ in LRS and *I*∝*V*^6.6^ in HRS) due to trap filling. Oxygen vacancies might serve as trap sites. Further, as the *I*-*V* curve of LRS is nonlinear, the oxygen vacancy conducting filament might not be dense; generally, ohmic conduction is observed in a thick and dense filament [28]. The switching mechanism can be attributed to the formation/rupture of the oxygen vacancy conducting filament upon the application of appropriate electric field.

**Figure 5:**
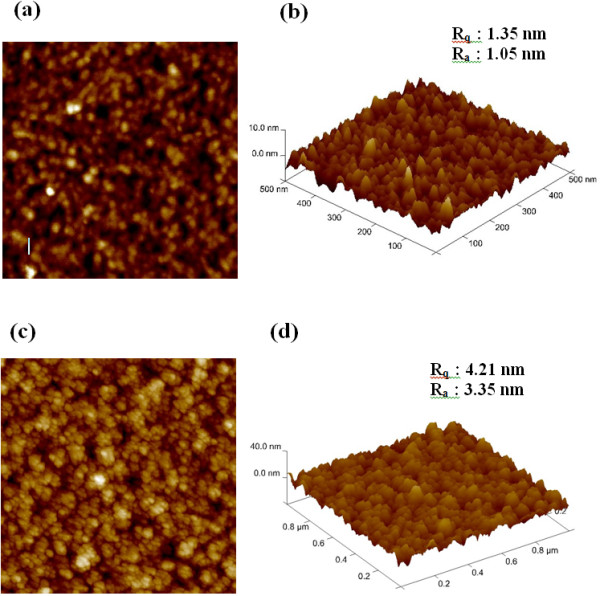
**Surface roughness by AFM. (a)** 2D and **(b)** 3D AFM images of the smooth surface, and **(c)** 2D and **(d)** 3D AFM images of the self-assembled nanotip W BE surface.

**Figure 6:**
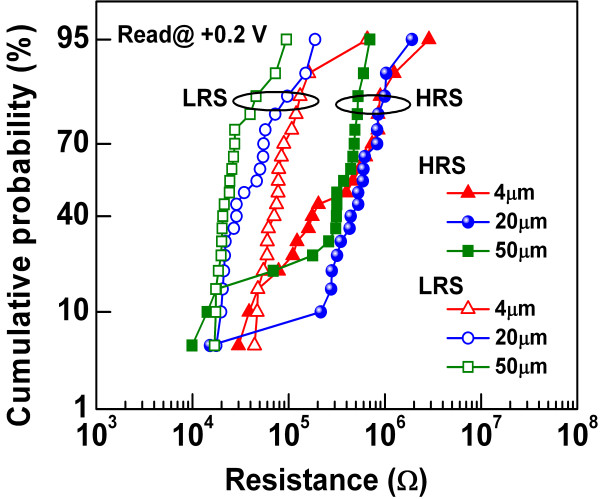
**Cumulative probability of HRS/LRS.** Cumulative probability of 4 × 4, 20 × 20, and 50 × 50 μm^2^ cross-point resistive switching memory devices.

**Figure 7:**
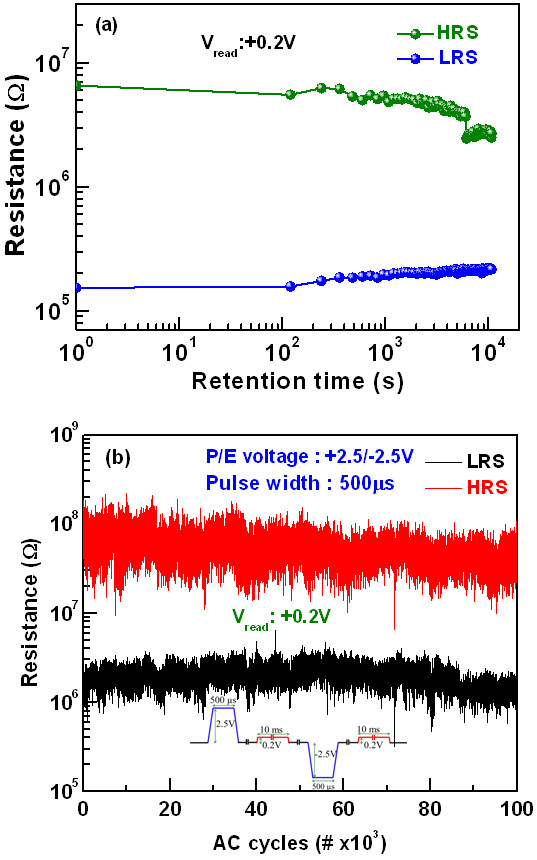
**Data retention and endurance. (a)** Good data retention and **(b)** excellent ac endurance with every cycle reading of >10^5^ are obtained. All switching devices have such a long endurance.

The improvement in the switching can be co-related with the surface morphology of the W bottom electrode observed in the AFM image, as shown in Figure 5. The enhancement of the electric field at the tips can help in controlled filament formation/rupture which leads to the uniformity in the switching parameters. Similar results are reported by Huang et al. [29]. These two types of BEs with different surface roughness were prepared by controlling the deposition method (sputtering or PECVD) and parameters such as power or working pressure during sputtering. The AFM images of smooth and nanotip BE surfaces are shown in Figure 5. Figure 5a,c shows two-dimensional (2D) or planeviews of surface roughness for the smooth and nanotip samples, respectively. Figure 5b,d shows 3D views of the smooth and nanotip samples, respectively. The average (*R*_a_) and root mean square (rms; *R*_q_) surface roughness values of smooth and nanotip BE surfaces are found to be 1.05 and 1.35 nm, and 3.35 and 4.21 nm, respectively. These self-assembled nanotips are observed from our W BE surface. Experimental data shows that the switching cycle uniformity and pulse endurance were greatly improved in the devices with nanotip BE surface. This is due to the controlled and easy formation/rupture of the conducting filament during switching owing to the enhanced electric field at the nanotips observed in the AFM image. Also, it is expected that the film will be more defective on the nanotip BE surface. Due to these reasons, the cross-point memory device shows almost forming-free or low-voltage operation. Figure 6 shows the device-to-device cumulative probability plot of LRS and HRS of cross-point memory devices with different sizes of 4 × 4, 20 × 20, and 50 × 50 μm^2^, respectively. More than 20 cross-points of each size have been measured randomly across the 4-in. wafer. Most of the devices show resistive switching with an HRS/LRS ratio of >10. The average resistance of LRS increases by decreasing the device size from 50 × 50 to 4 × 4 μm^2^. This might be due to the multifilament formation which is more probable when the device size is large, which is due to the nonuniform deposition of the switching layer on the sidewalls. It is expected that device-to-device uniformity can further be improved under a better facility. In order to confirm the nonvolatility of LRS and HRS, the resistance of both states is monitored with time and plotted in Figure 7a. The read voltage was +0.2 V. As can be seen, both LRS and HRS are fairly stable for more than 10^4^ s at room temperature. Figure 7b shows the ac endurance capability of our cross-point memory device. The device was successively programmed and erased at +2.5/−2.5 V with 500-μs pulse, respectively, and read after each program/erase event at +0.2 V, as schematically shown inside Figure 7b. The data of every such program/erase event is recorded and plotted. The read pulse width was 10 ms. Due to every cycle read, variation of HRS/LRS with cycle-to-cycle is observed, which is slight read disturb. Further study is necessary to overcome this problem. However, an excellent ac endurance of more than 10^5^ cycles is achieved. A high resistance value of LRS (approximately 1 MΩ) might be useful in fabricating large size arrays by suppressing the leakage current from unselected cells and reduce the active power consumption.

## Conclusions

Improvement in the resistive switching and self-compliance behaviors of a forming-free resistive memory stack of Ir/TaO_*x*_/W in a cross-point structure has been obtained. The cross-sectional TEM image confirms the amorphous TaO_*x*_/WO_*x*_ film. The AFM image shows the presence of nanotips on the W bottom electrode surface. The device has shown excellent switching uniformity during 100 consecutive dc sweeps with set/reset voltages of ±2.5 V and a resistance ratio of >100. The self-compliance behavior which comes from the bulk resistance of the stack shows the built-in capability of the device to minimize current overshoot during switching. The improvement in the switching is attributed to the formation of a defective switching layer and bottom electrode surface morphology with nanoscale tips which can enhance the electric field resulting in the uniform formation/rupture of the oxygen vacancy conducting filament. The device has exhibited an ac cycle endurance of >10^5^ cycles and a data retention of >10^4^ s. It is expected that this self-compliance, low-voltage-operated cross-point resistive memory device could be useful for the development of future nanoscale nonvolatile memory devices.

## Competing interests

The authors declare that they have no competing interests.

## Authors’ contributions

AP carried out the fabrication, measurement, and analysis of the cross-point memory devices, and he wrote the manuscript under the instruction of SM. DJ and SS measured the memory devices under the instruction of SM. All authors contributed to the revision of the manuscript, and they approved it for publication.
